# Right coronary artery supply to a cardiac mass

**DOI:** 10.1007/s12471-019-01319-7

**Published:** 2019-08-22

**Authors:** J. M. Zelis, N. Westphal

**Affiliations:** Department of Cardiology, Catharina Hospital Eindhoven, Eindhoven, The Netherlands

**Fig. 1:**
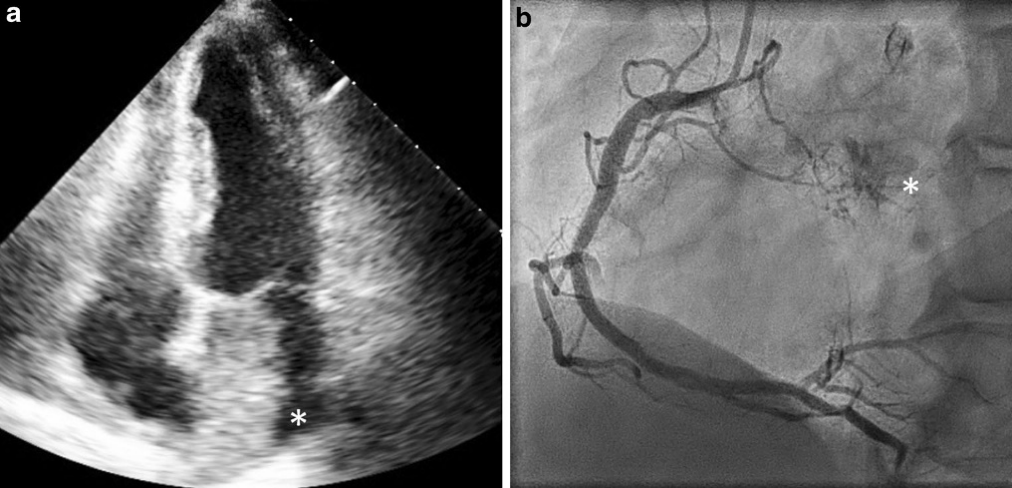
**a** Echocardiogram showing cardiac mass in left atrium. **b** Coronary angiogram showing supply to an extra-cardiac mass from the right coronary artery

A 73-year-old patient with previously percutaneously treated coronary artery disease and a history of smoking was referred for routine preoperative screening in preparation for a transurethral tumour resection. He reported dyspnoea. Physical examination revealed normal cardiac auscultation without signs of heart failure. Echocardiography identified a mass of 56 by 32 mm in the left atrium (Fig. 1a) near the anterior leaflet of the mitral valve attached to the atrial septum (*asterisk*). Subsequently, MRI revealed that the mass was a myxoma. Preoperative coronary angiography showed no significant coronary artery stenoses. When contrast was injected into the right coronary artery a clear side branch (Fig. 1b) with contrast blush to a structure in the left atrium was seen, corresponding to a left atrial tumour (*asterisk*). As is known from the literature, left atrial myxomas are often vascularised by the circumflex artery, closely followed by the right coronary artery [1, 2]. Complete tumour resection followed, which left the patient in good health.
